# Spatial-Aware and Load Balancing Distributed Data Partitioning Strategies for Content-Based Multimedia Retrieval

**DOI:** 10.21203/rs.3.rs-4973077/v1

**Published:** 2024-09-24

**Authors:** Gabriel Pereira, Willian Barreiros, Renato Ferreira, George Teodoro

**Affiliations:** 1 Department of Computer Science, Universidade Federal de Minas Gerais, Belo Horizonte, Minas Gerais, Brazil.

**Keywords:** Content-Based Multimedia Retrieval, Data Partitioning, Load Imbalance, Approximate Nearest Neighbors, Product Quantization, Distributed Computing, Similarity Search

## Abstract

Content-Based Multimedia Retrieval (CBMR) has become very popular in several applications, driven by the growing routine use of multimedia data. Since the datasets used in real-world applications are very large and descriptor’s dimensionality is high, querying is an expensive, albeit important functionality. Further, exact search is prohibitive in most cases, motivating the use of Approximate Nearest Neighbour Search (ANNS) algorithms, trading accuracy for performance. These have been mainly developed targeting a sequential execution in a single node. However, the large and increasing datasets used and the high query loads submitted to those systems typically surpass the memory and computing resources available in a single node. This motivated the development of parallel distributed memory ANNS solutions to meet the computing capabilities required by those applications. A common problem that must be handled when using distributed memory systems is data partitioning and its impact on load imbalance. Several data partitioning approaches have already been proposed, including elaborated spatial-aware strategies. However, little effort has been put into carefully analyzing the performance of those strategies at scale. Here, we evaluated the commonly used data partitioning strategies in ANNS and identified their limitations to propose a novel class of partitioning algorithms that can minimize load imbalance while improving data locality to attain high performance on the distributed memory search. Experimentally, we found that our proposed algorithms (SABBS and SABBSR) improved search performance by up to 1.64× compared to the best previous solution. In a distributed memory weak scaling evaluation, with up to 12 billion 128-dimensional descriptors and 60 compute nodes, the gains were maintained as the system scaled with our novel approaches. These results demonstrate the efficiency of our new algorithms for billion-scale ANNS and the importance of considering not only data locality but also data and load imbalance in the data partitioning.

## 1 Introduction

Content-Based Multimedia Retrieval (CBMR) tools and applications are already very popular and are still gaining traction in several domains. Some of these include automatic tagging of photos in social networks and image search engines [1, 2]. In these systems, multimedia objects (e.g. images and videos) are represented using high-dimensional feature vectors (descriptors), such as Scale Invariant Feature Transform (SIFT), GIST, and Deep Features [3, 4]. Although a CBMR usually consists of several steps, a core operation in these systems refers to the similarity search. This search consists of finding the descriptors in a database that are closest to a given query descriptor.

The exact k-nearest neighbors (k-NN) solution that exhaustively computes the distance from the query to all descriptors in the database is an impracticable strategy. Such an approach is too costly due to the increasingly large number of objects/descriptors indexed by a real-world system and their high dimensionality. This motivated the development of Approximate Nearest Neighbors Search (ANNS) solutions, which provide an accuracy for performance trade-off. Popular ANNS solutions include but are not limited to locality-sensitive hashing (LSH) [5], the fast library for approximate nearest neighbors (FLANN) [6], and the Inverted File system with Asymmetric Distance Computation (IVFADC) [7]. These ANNS algorithms organize the dataset of descriptors into buckets according to their spatial location and restrict the search to a limited number of buckets. IVFADC additionally employs a product quantization on top of the descriptors to drastically reduce their dimensionality. This improves the memory requirements to store the index and search speed since distance computation is carried out in the quantized (low-dimensional) space. Thus, IVFADC stands as one of the most competitive solutions in the literature and it is especially efficient and effective when large datasets are indexed.

While ANNS indexing strategies have significantly improved the performance of CBMR systems, those solutions were originally proposed focusing on their sequential execution on a single node. However, the memory and computation demands of modern large-scale applications quickly exceed the capabilities of a single compute node. In turn, this motivated the development of distributed memory solutions that are able to jointly use the computing power and memory storage of clusters of nodes to execute efficiently ANNS search in large-scale datasets [6, 8, 9, 10, 11, 12, 13, 14, 15].

Those distributed memory ANNS solutions have leveraged different programming tools, indexing algorithms, and parallelization strategies. In this work, we focus on evaluating existing strategies and proposing new approaches. The most common strategies used in the literature are: the Data Equal Split (DES) and Bucket Equal Split (BES). DES evenly distributes descriptors among nodes for indexing and, consequently, must probe all nodes during a query search. BES distributes partitions of the dataset (buckets) defined by the ANNS algorithm among nodes for indexing. Thus, during the search only nodes containing buckets that are close to the query descriptor are involved in the search. More recently, we proposed a novel parallelization strategy called Spatial Aware Bucket Equal Split (SABES) [14]. It extends BES by distributing buckets to nodes while trying to allocate a set of buckets close in the space to the same node. Thus, during the search, it is expected that fewer nodes are visited on a query when compared to BES since buckets used to answer a query in ANNS algorithms are also typically close in the space. As a consequence, ANNS is cheaper in SABES, outperforming BES and DES both in terms of performance and scalability in several settings.

However, we noticed that the SABES data partitioning, although spatially efficient, can lead to data imbalance among nodes. A subset of the nodes in the distributed system may be in charge of storing and searching in a larger number of descriptors, resulting in an imbalanced execution load with few nodes becoming the bottleneck of the overall search. To address this issue, in this work, we propose a novel class of data partitioning strategies that benefit from the spatial awareness collocation of buckets introduced in SABES, while at the same time reducing/limiting the load imbalance among nodes. The proposed strategies are: Spatial-Aware Bucket Balanced Split (SABBS) and Spatial-Aware Bucket Balanced Split with Relevance (SABBSR). SABBS constrains the imbalance by limiting the number of buckets assigned to a node using a threshold with respect to the number of descriptors assigned to them. SABBSR is a more sophisticated strategy that takes into consideration the number of points in a bucket and also how frequently a bucket is queried (bucket/centroid relevance) to measure this impact on processing costs. Using this new metric, SABBSR groups centroids as in SABES, but avoids imbalance by computing the expected load of each node. Our contributions can be summarized as follows.

- We evaluated state-of-the-art data partitioning strategies using distributed memory parallelization of ANNS to identify their limitations;
- We proposed and implemented new approaches for ANNS data partitioning (SABBS and SABBSR) that improve on the existing literature. Our strategies consider both data spatial locality and load imbalance generated by the partitioning process.
- We carried out a number of experimental analyses varying important parameters of the algorithm. The experiments have shown different performance gains of the SABBS and SABBSR compared to previous strategies. For instance, when using a small number of coarse centroids, SABBSR attains better gains with up to 1.64× improvement vs the best available approach. Also, as the number of buckets visited (w) increases the SABBSR also improves performance.
- We conducted an experimental evaluation of weak scaling using a maximum of 60 computing nodes (60 workers), scaling both dataset size and computing resources proportionally, reaching the scale of 12 billion SIFT vectors. The analysis has shown that our SABBSR attains consistent gains over all strategies as the system is scaled.

The remainder of this document is organized as follows. The related work on distributed memory ANNS is discussed in Section 2. IVFADC is detailed in Section 3 as is the ANNS indexing algorithm parallelized in our work. Further, Section 4 presents the framework employed to parallelize IVFADC for distributed memory machines along with existing data partition strategies. We then describe the novel data partition approaches proposed in this work in Section 5, whereas the experimental results are discussed in Section 6. Finally, conclusions and future directions are available in Section 7.

## 2 Related Work

The Approximate Nearest Neighbour Search problem (ANNS) has been extensively studied in the last decades. Among strategies available in the literature we highlight three most successful classes of solutions. These approaches provide solutions for ANNS in high-dimensional spaces using large datasets on the scale of millions to billions of features. Graph-based approaches have the most abstract set of models, which results in a diverse set of solutions. Its modeling allows the use of extensively researched graph algorithms. More recently, it has focused on processing datasets larger than memory through indexing, pruning, and optimized graph-building algorithms [16]. Another interesting approach is the use of Locality-Sensitive Hashing algorithms (LSH) [5], which uses hashing functions with higher collision rates for similar data points to organize data points into buckets. By employing multiple hashing functions during the hashing it asserts that only points with a high probability of being close in the space are stored in the same bucket or nearest neighbors candidate list. However, to return adequate search results it is usually necessary to employ a large number of hash tables, whose sizes also increase with the dataset size. This results in a large memory footprint and can be cumbersome to maintain in-memory. Finally, Product-Quantization-based approaches (PQ) [7] encode vectors (points on the dataset or the query itself) using quantizing representations. This alleviates the problems with the curse of dimensionality [17] by reducing the data space dimensionality. Additionally, the encoded representations can be used in conjunction with other data structures to prune the search to a small partition of the dataset and, as a consequence, to quickly answer queries.

Although many recent publications carefully survey the advances of each approach [16, 18, 19], we have selected some recent relevant works to compare our solution with. When surveying ANNS publications we found two main types of works: encoder-based works and structure-based works. The first type focuses on providing efficient ways to represent high-dimensional data, which can improve accuracy or reduce memory footprint. The second type approaches the problem of organizing, storing, and querying the data. The related works here revised, as our proposed solutions, target the second type of work. Further, we also restrict the works to those approaching the distributed memory execution of ANNS.

Our evaluation was based on the three main algorithms discussed above and while some techniques applied to these works can be employed to solve other distributed computing problems or general ANNS problems, we focused on the original context in which these were implemented and evaluated. Following, we evaluated how indexing data structures are stored and accessed, which could be in-memory or in secondary storage. The advantage of using in-memory solutions is the reduced data access overheads (which can be exacerbated if secondary storage is not insite [25]). However, Out-Of-Core (OOC) solutions allow indexing larger datasets, which may be necessary depending on the computing platform available and the indexing algorithm used. Finally, we evaluated how data is partitioned to be distributed among compute nodes based on the following sub-classification: partitioning considers the size of the partitions and explicitly attempts to balance them across resources (Data-Balancing Aware), focuses on optimizing the proximity of data points/buckets assigned to a node (Spatial Aware), and attempt to balance the load when distributing the dataset (Load Balancing Aware). It is worth noting that the Data-Balancing feature can be trivially implemented by itself through Data-Equal Partitioning (DES, see Section 4.2). This is a common case for many partitioners [22, 23, 24, 25] given the load imbalance improvements and ease of implementation. On the opposite end, it is also possible to focus entirely on spatial awareness at the cost of data balancing, which is the case for [21, 14]. A summary of these features is presented in Table 1 for the closest works.

LSH solutions were important in making the field more attractive with their high accuracy and provable guarantees of quality. The work of [20] proposed a distributed memory LSH implementation. Through the use of a Delta Table, which is periodically merged into the existing data structure, new values can be dynamically (or at run-time) inserted into the global index kept in-memory. Data is initially evenly partitioned among compute nodes in a round-robin manner. However, for their application, old (less relevant) data points can be retired to make space for new descriptors. To avoid using a timestamp and keep a garbage-collector-like process running in the background a subset of *M* nodes (*M* less than the total number of nodes) is used for insertions. Each subset of *M* nodes is filled one at a time. When all nodes are full the oldest *M* subset of nodes can be retired. This can lead to an imbalanced data distribution among compute nodes. The work [21] proposed a Shuffle-efficient search (SES) to execute LSH in a distributed environment. After the initial indexing of equally distributed descriptors, these are shuffled between worker processes to improve locality within workers with no guarantee of data balance. Further queries are processed only by workers with partitions close to the query object. In [22], data is split equally between nodes, and each query is forwarded to every node. The partial results of each node are then merged and returned to a central node. Data distribution among nodes is random and data partitions are independently indexed at each node.

FLANN [23] introduces the novel priority k-means tree. The index is created by clustering points around an initial set of centroids, which are then used to partition once again the domain in a hierarchical fashion. Each sub-region is recursively divided into *K* regions, stopping when a partition with less than *K* points is reached. Index data is split equally among compute nodes and, thus, queries are processed by all nodes. At the end, node-local results are merged by a master process. Pyramid [24] is a distributed framework that implements the graph-based Hierarchical Navigable Small-World (HNSW) algorithm. Instead of creating multiple HNSW indices for evenly distributing data points among nodes, Pyramid generates a global smaller meta-HNSW index. From each endpoint of this meta-HNSW, sub-HNSW indices are generated and distributed among nodes. Thus, after an initial search of the query on the meta-HNSW, only sub-HNSW indices close to the query are used. When indexing, Pyramid attempts to balance the amount of data between nodes and it also replicates sub-HNSW indices to allow fault tolerance. However, graph-based solutions are very memory-demanding and, as such, not adequate for large-scale datasets.

SPANN [25] proposes an inverted index memory/disk hybrid solution. The initial dataset is evenly split into so-called posting lists, each with a given centroid. A query point is searched among all posting list centroids in memory. A defined *K* closest centroids have their corresponding posting lists searched for the nearest candidates to the query point. Posting lists can remain in memory for memory-constrained environments and are retrieved when not available in main memory. This approach generates a large number of posting lists to reduce the disk access penalty for out-of-core posting lists. Further, it can also configure the value of *K* to deal with different resource budgets. Finally, Spatial-Aware Bucket Equal Split (SABES) [14] presented in our previous work was an attempt to improve search performance through a data partitioner that improved data locality. However, as briefly discussed, the load and data imbalance could become an issue with this strategy as it only considers locality when computing the partitioning. However, the concepts and interesting performance improvements with SABES were fundamental motivations for this work, since the newly proposed approaches extend SABES.

## 3 Inverted File system with Asymmetric Distance Computation (IVFADC)

IVFADC is built on quantization to reduce the data dimensionality and inverted files to prune the search to a subset of the database. Quantization can be more formally defined as follows: given a vector $x\in R^{D}$ with D dimensions, the quantization function q maps x to a q(x) vector with $D^{\prime }\;<\;D$ dimensions. For a finite index set $I\;\in \;\{0...D^{\prime }\;-\;1\}$, we define the codebook $C=\left\{c_{i};i\in D^{\prime }\right\}$ comprised of k centroids $c_{i}$ that represent the lower dimensional representation of x:q(x)∈C. In this context, larger codebook sizes would be required to attain a high quantization quality. However, this can become impractical since large codebooks require more time to compute the quantization and more storage space.

These requirements for larger codebooks are addressed in IFVADC with the use of product quantization. In this approach, vector x is split into m disjoint subvectors $u_{j};j\in 1\ldots m$ with a dimensionality $D^{*}=D/m$. The quantization of vector x can then be defined as:

(1) $$\begin{array}{rr} & \underset{u_{1}(x)}{\underbrace{x_{1},\ldots ,x_{D^{*}}}},\ldots ,\underset{u_{m}(x)}{\underbrace{x_{D-D^{*}+1},\ldots ,x_{D}}}\\ & \;\to q_{1}\left(u_{1}(x)\right),\ldots ,q_{m}\left(u_{m}(x)\right) \end{array}$$

Here, $q_{j}$ is a less complex subquantizer for the j^*th*^ subvector when compared with a quantizer q for the entire x vector. The new codebook C is then a Cartesian product of the m composing codebooks $C_{j}$ for each subvector $u_{j}$:

(2) $$C=C_{1}\times \ldots \times C_{m}$$

This allows building a larger codebook with less complex quantizers and reduced memory cost. For instance, assuming the use of k centroids in each subvector, the codebook C would contain $k^{m}$ quantization combinations requiring only m simple quantizers for k possible indexes.

Once the descriptors are quantized and stored in the database the search for a query still needs to compute distances between the query and quantized descriptors. An efficient approach to perform this operation was also proposed with IVFADC and is called Asymmetric Distance Computation (ADC) [7]. It calculates the distance from a non-encoded (non-quantized) query x to q(y), an already quantized vector in the database for document y. It was observed empirically that distances computed using the non-quantized are more precise than when the quantized query is employed. The ADC d(x,y) is defined as follows:

(3) $$d\left(x,y\right)\approx d\left(x,q\left(y\right)\right)=\sqrt{\sum_{j}^{m}d{\left(u_{j}\left(x\right),q_{j}\left(u_{j}\left(y\right)\right)\right)}^{2}}$$

Although ADC is less compute-demanding in quantized spaces, it is still necessary to optimize search by reducing (or pruning) the search space. Yet, to prune the search space and avoid computing, the distance of a query to all points in the database a data structure called inverted file is used. Nominally this strategy uses ADC with the Inverted File (IVFADC). Thus, the database Y can be indexed through a set of inverted file entries $L_{1}\ldots L_{k^{\prime }}$. Each list $L_{i}$ contains elements y from Y such that $q(y)=c_{i}$. Here, $c_{i}$ is a centroid from the set $C_{c}$, which consists of k′ coarse centroids determined by the quantization function $q_{c}$.

**Figure 1:**
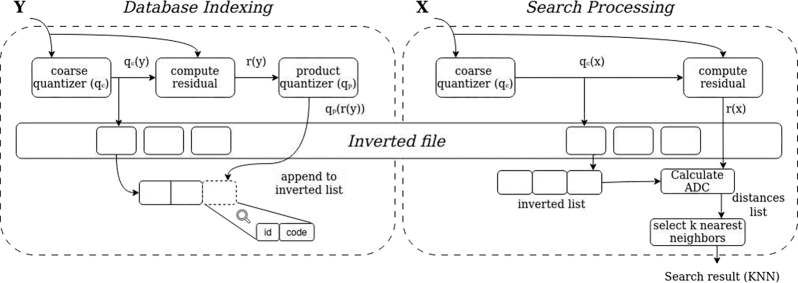
Representation of Database Indexing (left) and Search Processing (right) in an *Inverted File with Asymmetric Distance Computation* (IVFADC). In the indexing phase, the quantization of the descriptor is performed with its residual being stored in the inverted list. During the search phase, the query is quantized to determine close inverted list entries. Then its distance to the descriptors in the relevant inverted lists is calculated, determining the k nearest neighbors.

The indexing and searching phases can be seen in Figure 1. In the indexing phase, each vector y is quantized for $q_{c}(y)$ and the residual of y is computed as $r(y)=y-q_{c}(y)$. Then, the residual is quantized for $q_{p}(r(y))$, which, in the context of the product quantizer, means assigning $u_{j}(y)$ to $q_{j}\left(u_{j}(y)\right)$, for j=1...m. At the end, the new entry is added to the corresponding inverted list $q_{c}(y)$, containing the vector identifier and its index.

For the searching phase, the query vector x is quantized to its w nearest neighbors in codebook $q_{c}$. Considering r(x) the residual values associated with each w assignment, the two next steps, which are performed in all w assignments, are: (i) compute square distance $d{\left(u_{j}(r(x)),c_{j,i}\right)}^{2}$ for each subquantizer j and each of its centroids $c_{j,i}$, and (ii) compute square distance from r(x) to all indexed vectors of the inverted list by using the subvector-to-centroid distances computed before. Finally, the k nearest neighbors from x are chosen based on the estimated distances. In addition, w closest lists are visited given that the k nearest neighbors may not necessarily be in the nearest inverted list.

## 4 Distributed Memory IVFADC Parallelization

This section presents the distributed memory system used for parallelization of IVFADC (Section 4.1) and the data partitioning strategies employed in the previous works (Section 4.2).

### 4.1 Distributed Memory Parallelization Architecture

The parallelization employed here is based on the system design proposed in [26]. It decomposes the Approximate Nearest Neighbour (ANN) indexing into a workflow of data processing stages organized into two phases: Index Building and Searching. These phases share stages and the communication among them is carried out using the Message Passing Interface (MPI) [27]. In this architecture, each stage may be replicated as many times as necessary, which allows for the workflow to balance the amount of computing/memory used by each of them and, consequently, to avoid specific stages becoming bottlenecks of the workflow.

**Figure 2:**
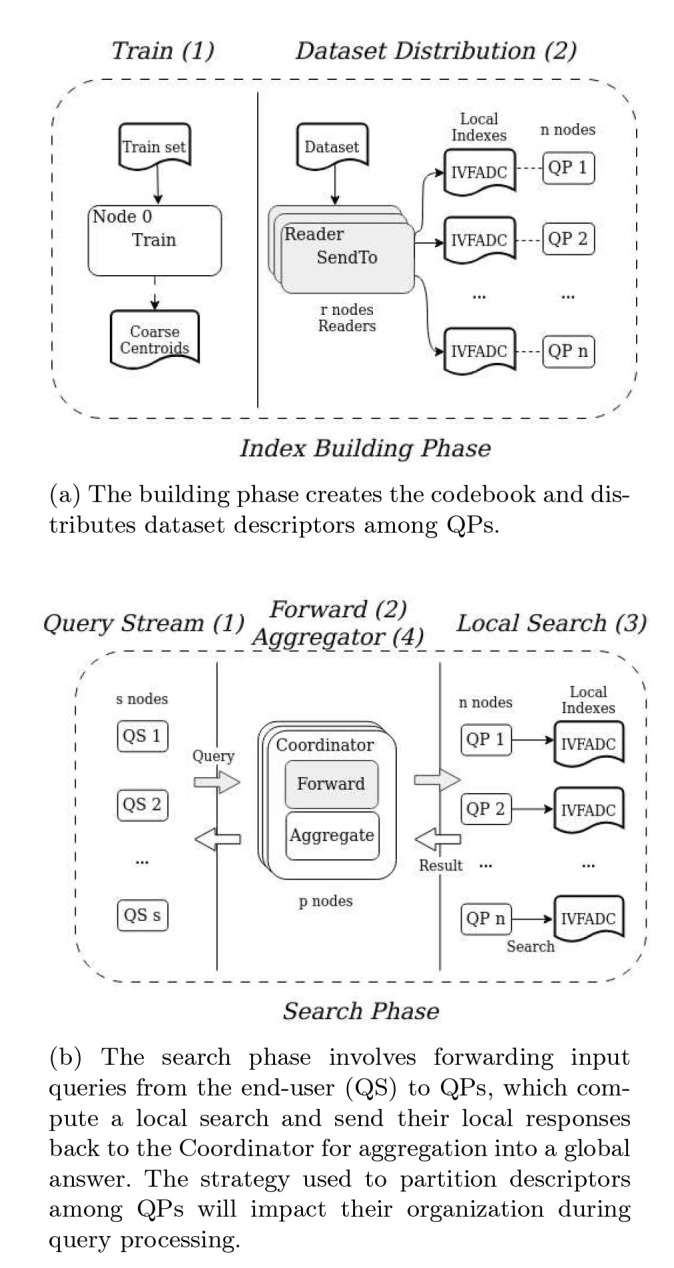
Two phases of the distributed memory indexing and searching using IVFADC: (i) Building Index; and (i) Searching. The modules *SendTo* along with *Forward* are customized to implement different data partitioning.

These phases of the distributed execution are presented in Figure 2 including stages used in each of them. The first phase, index building, creates a set of centroids (Coarse Centroids $C_{c}$) with the *k-means* algorithm using a subset of the dataset. This is typically computed off-line and when pre-computed will consist of just reading centroids from a file. The actual indexing of databases will use two stage process types: (i) Readers (Re) and (ii) Query Processors (QPs). The Readers are in charge of reading portions of the database, quantizing descriptors, and sending the quantized data to the QP(s) instance(s) in charge of indexing each data element. The mapping of a descriptor to the QP that stores it is implemented differently according to the data partition strategy employed and, in our system, it is computed in the *sendTo* operation (highlighted in gray in Figure 2a). As such, this function must be rewritten according to the partitioning strategy used. The description in the rest of this section assumes, for simplicity, that descriptors are distributed in a round-robin fashion among QPs. The next subsection describes how it is modified to implement other data partitioning strategies.

The search phase involves three types of stages (Figure 2b: (i) Query Stream (QS), (ii) Coordinators (Co), and (iii) Query Processor (QP). The first simulates user query submissions and sends the queries to the Coordinators. In the Coordinators, the query is forwarded to all QPs to perform the search, since the data is distributed in a round-robin among QPs (for data distribution used in this example). Further, the coordinator receives local nearest neighbors from those QPs to create the global query response. The forward operation (highlighted in gray in the Coordinator) receives each query and is responsible for routing it to specific instances of QP according to the data partition used. The Co processes each query by evaluating its distance to all centroids, with the w closest centroids selected, and QPs responsible for storing those centroids receive the forwarded query. Since these strategies directly impact data partitioning, the *forward* operation is also unique for each partitioning strategy along with the *sendTo* operation. Each QP receives a query and performs a search within its data partition using the regular IVFADC algorithm. The results are sent to the Co that aggregates the QPs results for each query. An identifier is assigned to each query, allowing the QPs to determine the Co instance responsible for a query.

The local search within the Query Processor nodes is carried out using two components: (i) Query Receiver/Result Sender thread responsible for communicating with the Coordinators; and (ii) Local Search, a multi-threaded execution flow that performs IVFADC search in the local dataset partition. Multiple queries can be executed concurrently with each thread handling one query. The k-nearest neighbors found in the search are sent to the Coordinator responsible for the query. The multi-threaded QP allows better use of compute resources and minimizes the number of data partitions in the distributed memory system. Only one QP can be instantiated per computing node and, as such, a single partition per node is required. In the following section, we describe how state-of-the-art data partitioning strategies are implemented in this framework.

### 4.2 Previous Data Partitioning Strategies

This section presents the data partitioning strategies that are currently employed in distributed memory ANN parallelization and describe how they are implemented in our framework, which consists of providing versions of the *sendTo* and *forward* operations are described in the previous section.

#### Data Equal Split (DES):

During the index building phase, DES distributes the input data feature vectors evenly among the QP processes in a round-robin manner (*sendTo*). As a result, each QP possesses a complete IVF structure containing all centroids (entries), while the actual data (descriptors) are partitioned among the QPs. To ensure all nearest coarse centroids lists are fully accessed during the search phase, queries must be broadcasted from the Coordinators to all QPs (forward). This approach has been utilized in various prior studies. DES is presented in Algorithm 1 with the pseudo-code for *sendTo* and forward functions. The sendTo function decides the QP instance to store object y by computing a modulo operation of a counter (or object id) with the number n of QP instances (line 2). Further, when a query x is received it must be sent to every QP instance. Thus, its *forward* operation returns the full list of n QPs available.

**Algorithm 1:** Data Equal Split (DES).

1:	**function** sendTo(y,n)
2:	ret←next **mod** n
3:	next←next+1
4:	**return** ret
5:	**end function**
6:	**function** forward(x,w,n)
7:	target←[]
8:	**for each** i∈0…n−1 **do**
9:	target←target∪i
10:	**end for**
11:	**return** target
12:	**end function**

#### Bucket Equal Split (BES):

ANN indexing algorithms organize feature vectors into data buckets where objects in the same bucket are expected to be spatially close. This organization is used by BES which distributes buckets of elements among QPs. Thus, only QPs storing a bucket of interest (used to answer a query) are utilized during query processing. This reduces communication and startup overheads during the search as compared to DES. For IVFADC this results in a distribution of inverted file structure (or its entries) QPs, ensuring all points in an entry reside in one QP. This partitioning is implemented by the function *sendTo* in Algorithm 2. It first computes the closest coarse centroid k′ to an object y to be indexed in line 2 and then assigns that object (and others quantized to the same centroid) to a specific QP instance in line 3. As such, coarse centroids and their respective data buckets are distributed in a round-robin fashion among QPs, with each QP storing $\left|C_{c}\right|/|QP|$ centroids. During the search, BES routes queries only to QPs holding its w closest centroids. Thus, as presented in Algorithm 2, the closest centroids to the query are computed (line 6), each of them is mapped to a QP instance (line 8), and the resulting set of QPs storing those centroids is returned.

**Algorithm 2:** Bucket Equal Split (BES).

1:	**function** sendTo(y,c)
2:	$k^{\prime }\leftarrow q_{c}(y)$
3:	$target\leftarrow k^{\prime }$ **mod** n
4:	**return** target
5:	**end function**
6:	**function** forward(x,w,n)
7:	$nnc\leftarrow kNN\left(C_{c},x,w\right)$
8:	**for each** i∈1…w **do**
9:	$target\leftarrow \;target\cup (nnc\left[i\right]\;mod\;n)$
10:	**end for**
11:	**return** target
12:	**end function**

#### Spatial-Aware Bucket Equal Split (SABES):

SABES [26] extends the concepts of BES with an additional spatial-aware distribution of centroids (or buckets), where coarse centroids close in space are assigned to the same QP. This strategy aims to reduce the number of QP instances required to answer a query. As a query visits the w closest centroids or inverted list entries, the idea is that organizing centroids near in space into the same QP increases the likelihood of finding the w closest centroids within a smaller number of QP instances when compared to BES. In SABES, coarse centroids $C_{c}$ are clustered into n regions R using the k-*means* algorithm (line 2 of Algorithm 3), where n is the number of QP instances and each $R_{i}$ is assigned to $QP_{i}$. This is computed by the *regionConstruction* function and is executed before any data partitioning is employed. As discussed, it creates a mapping from the coarse centroid and the QP in which it is stored. Afterward, in *sendTo* (Algorithm 3) the data point to be indexed is quantized to k′ (line 5), and the corresponding region i is retrieved (line 6). Further, during the search, the forward function computes the w closest coarse centroids as in SABES (line 9 of Algorithm 3) and, for each of them, the corresponding region is retrieved and added to the set of QPs that should process that query (line 11).

**Algorithm 3:** Spatial-Aware Bucket Equal Split (SABES).

1:	**function** constructRegion($C_{c},\;n$)
2:	$R\leftarrow \mathrm{k-means}(C_{c},\;n)$
3:	**return** R
4:	**end function**
5:	**function** sendTo(y,n)
6:	$k^{\prime }\leftarrow q_{c}(y)$
7:	$\text{target}\leftarrow getRegion\left(R,k^{\prime }\right)$
8:	**return** target
9:	**end function**
10:	**function** forward (x,w,n)
11:	$nnc\leftarrow kNN\left(C_{c},x,w\right)$
12:	**for each** i∈1…w **do**
13:	target←target∪getRegion(R,nnc[i])
14:	**end for**
15:	**return** target
16:	**end function**

#### KD-Tree:

The KD-Tree data structure supports multi-dimensional partitioning and is relatively well-known. To implement it here, we have used the *nanoflann* library [28] which is widely used due to its efficiency. It constructs trees rapidly and employs dimension-wise splitting, selecting the most representative dimension at each separation. Partitioning using KD-Tree involves distributing the C centroids of our target database among n query processors. A KD-Tree structure is constructed using the centroids as input and using the leaf nodes to generate N groups of centroids. The *nanoflann* allows us to set a maximum number of centroids per leaf. To ensure at least n leaf nodes are generated, we configured KD-Tree with a maximum of C/n centroids per leaf. This guarantees that the number of leaf nodes (L) will be greater than or equal to the number of query processors (n).

The data partition process that follows the tree construction can lead to two scenarios according to the number of leaves L. In the ideal scenario, L is the same as the number of QPs (n) and each leaf node, which contains a certain number of centroids, is assigned to a QP. In the most common case, L exceeds n. Thus, the leaves are sorted by a non-increasing order and are allocated to QPs in sequence. To address the imbalance, the L−n leaves with the fewest centroids assigned to the QP with the smallest number of centroids. This heuristic helps to balance the dataset size across the Query Processors.

**Algorithm 4:** Building KD-Tree Regions.

1:	**function** constructRegion($C_{c},\;n$)
2:	$\mathrm{tree}\;\leftarrow \;\text{buildTree}(C_{c},\;n)$
3:	sort(tree.leaves())
4:	**for each** i∈1…n **do**
5:	regions[i]←tree.leaves[i]
6:	**end for**
7:	**for each** i∈n…size(tree.leaves) **do**
8:	region←selectLighterRegion()
9:	region←region∪tree.leaves[i]
10:	**end for**
11:	**return** regions
12:	**end function**

#### Complexity:

The DES strategy broadcasts each query to all query processors. Therefore, determining the destination has a time complexity of 𝒪(1), and messages are received by at most n query processor processes. In both BES and SABES, as with KD-Tree, an offline map of centroids per region is established and used in the *sendTo* and *forward functions*. These strategies employ k-nearest neighbors (kNN) on coarse centroids during *sendTo*, which entails 𝒪(|Cc|*D+|Cc|*log(w)) to retrieve the nearest set of coarse centroids. These centroids are mapped to the corresponding query processor instances in a subsequent loop. This loop performs w dictionary lookups with 𝒪(1) complexity each. Thus, the overall complexity during forwarding in both algorithms is 𝒪(|Cc|*D+|Cc|*log(w)+w), and messages are delivered to 𝒪(w) query processor instances.

## 5 Spatial-Aware and Load Balancing Data Partitioning Strategies

This section presents our novel data partitioning strategies for parallel and distributed memory execution of ANNS. As discussed in the previous section, several approaches were presented in the literature, such as DES, BES, and SABES. Among those strategies, SABES typically attained the best performance for several configurations, thanks to its spatial-aware data distribution that groups spatially close clusters in the same compute node. This results in better memory locality and, as a consequence, reduces the number of nodes involved in query computation and network traffic.

However, SABES only considers data locality during partitioning. Thus, its data distribution may lead to a significant load imbalance among compute nodes, limiting gains at scale. The imbalance is a result of not considering the expected processing (and memory) costs of a partition when organizing the data. For instance, SABES groups buckets (clusters in IVFADC) together at the same node without considering either (i) the number of data objects stored in a bucket or (ii) the frequency with which a bucket is consulted when executing a given query workload. Both aspects are very relevant in a data distribution as the first results in large memory demands and data processing costs when probing a data bucket, the second indicates that buckets more frequently used to process queries demand more computing power. The next sections present two novel data partitioning strategies that consider these aspects.

### 5.1 Spatial-Aware Bucket Balanced Split (SABBS)

This section presents our SABBS partitioning algorithm, developed as an extension to the concepts of Spatial Awareness employed in SABES. As such, it also tries to assign together to the same node (QP) data buckets that are spatially near. However, in SABBS, the concepts of data balancing are considered, trading some of the gains in locality for partitions with reduced imbalance. To this end, we define a ceiling value representing the number of data elements that should ideally be stored in each QP instance. This value, defined here as the threshold, is the ratio of the dataset size (|δ|) and the number of QP instances used in the execution: threshold=|δ|/|QP|. Thus, as buckets are distributed among QPs, our SABBS approach uses this threshold limiting factor to make regions or groups of buckets assigned to a QP as close as possible to a balanced distribution (mean value).

**Algorithm 5:** Building Regions for SABBS.

1:	**function** constructRegion($C_{c},\;n$)
2:	threshold←getDataSetSize()/n
3:	$\mathrm{kMeansRegions}\;\leftarrow \;\text{k-means}(C_{c},\;n)$
4:	**for each** i∈0…n **do**
5:	balancedRegions[i]←[]
6:	**end for**
7:	**for each** $c\;\in \;C_{c}$ **do**
8:	closestRegions←k-NN(n,c,kMeansRegions.centroids)
9:	**for each** i∈0…n **do**
10:	**if** balancedRegions[closestRegions[i]].size()+c.size()≤threshold **then**
11:	r←i
12:	**break**
13:	**end if**
14:	**end for**
15:	**if** r<n **then**
16:	balancedRegions[closestRegions[r]].insert(c)
17:	**else**
18:	balancedRegions[closestRegions[0]].insert(c)
19:	**end if**
20:	**end for**
21:	**return** balancedRegions
22:	**end function**

As previously mentioned, SABBS is an extension to SABES and, as such, it uses a similar approach as presented in Algorithm 5. The distinction is that regions are created differently considering spatial location and load balancing restrictions. Thus, SABBS implements a novel region construction strategy delineated in Algorithm 5, whereas the *sentTo* and *forward* are the same as used in SABES. The SABBS *regionConstruction* function receives the coarse centroids set $\left(C_{c}\right)$ and the number of partitions that should be created (n) which is the number of QP instances used. It then computes the threshold value (line 2) or the ideal number of data points to store in each partition. Further, a k-means clustering is employed on top of the coarse centroids to group them into n regions called k-*meansRegions* (line 3) without evaluating the number of data objects in a region.

The construction of the regions considering their sizes takes place with the loop shown in lines 7– 20. Here, the algorithm iterates over coarse centroids assigning them to their final region. For each coarse centroid c it computes an ordered list of the closest regions to c (considering the regions centroids), which is carried out using a k-NN with size n (line 8). After that point, the loop encompassing lines 6 to 8 will iterate over each *balancedRegions* set to find a region in which the bucket represented by coarse centroid c could be inserted. At this point it checks, from the closest region to the farthest, if inserting c and its attached points in that region would not make the region larger than the threshold (line 10). If this is the case the loop breaks. Otherwise, the loop continues until a region that fits the current threshold is found or all regions are scanned. After this loop, c is inserted either in the closest region i in which it fits (line 16) or in the closest regions among all in case none of the regions can fit c without breaking the balancing controlled by threshold (line 18). Finally, when all centroids are assigned to a region, the balanced regions are returned.

The algorithm then builds a routing table using this information in which each line is associated with a centroid and contains the region in which it is stored. This table is used in the *sendTo* and *forward* functions to retrieve the machine (QP instance(s)) that should process a query in 𝒪(1).

**Figure 3:**
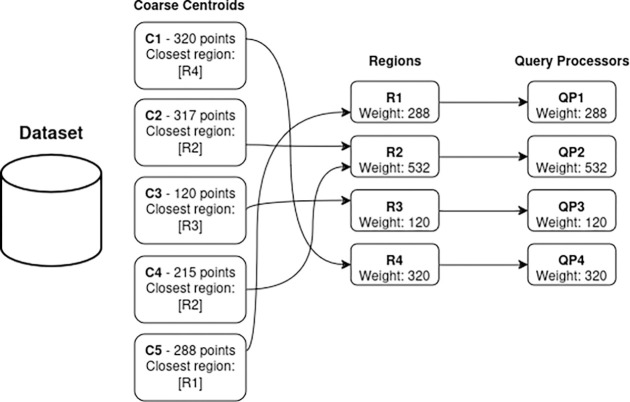
SABES data partitioning example. Centroids are assigned to their nearest region. This process may generate imbalanced regions. Notably, region **R2** is heavier than the other regions, resulting in an imbalance among QPs.

**Figure 4:**
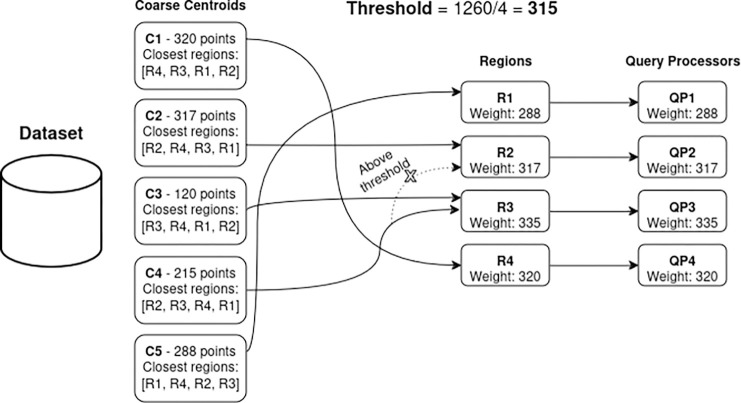
SABBS data partitioning example. Centroids are assigned to their nearest region that has not yet reached the weight threshold. The image illustrates a scenario where centroid **C4**, initially intended for region **R2**, is assigned to its second closest region (**R3**) because the first region had already exceeded the expected threshold.

Figures 3 and 4 illustrate the centroid assignment for both SABES and SABBS strategies. The dataset is represented by coarse centroids or data buckets (C1. . .C5) with the number of data points they store. In SABES (Figure 3), k-means clustering is used to compute |QPs| regions based on the centroids. Each centroid is then assigned to its nearest region, and each region is allocated to a QP. The weight of a region is defined by the number of points (descriptors) it stores. Consequently, the QP that receives a region inherits its weight (or the sum of weights from the centroid subset). This method can result in imbalanced QP data partitioning as shown in the example where QP2 has 532 points and QP3 contains 120 points.

In SABBS (4), the k-means clustering is again used based on the coarse centroids. Then, a *threshold* is defined as the total number of descriptors divided by the number of QPs. For each centroid, the algorithm lists the closest regions and prioritizes the assignment of centroids to the nearest region that does not exceed the *threshold*. As illustrated, the algorithm would try to assign centroid **C4** to region **R2**, but it is not allowed because the size of this region is larger than the threshold (317 points vs a threshold of 315). Thus, **C4** is instead assigned to **R3** which is the second closest region. Finally, each region is allocated to a QP. This method results in more balanced QP weights since the number of descriptors (weight) in each region tends to be closer to the mean/threshold.

### 5.2 Spatial-Aware Bucket Balanced Split with Relevance (SABBSR)

This section presents the SABBSR data partitioning algorithm, proposed to incorporate the notion of the relevance or computational load of data buckets (centroids). The concept of relevance was created to incorporate the aspect that, as in other retrieval applications, particular data buckets or centroids are accessed more frequently than others due to their content. As a consequence, when assigning centroids to regions and QP instances, relevance should also be considered along with the size of the data stored on a partition since it plays a crucial role in defining the processing cost of partitions.

In our approach, the relevance is measured as the fraction of queries that use a centroid and is calculated in an offline profiling stage in which a separate query workload is employed. It is important to highlight that the measurement of centroid relevance does not require the complete processing of a query, but only the computation of the w nearest coarse centroids. This is an important feature because the relevance must be computed before the index is distributed among QPs, since this metric is used in the partitioning. We have experimentally validated the divergence in centroid relevance in Section 6.2. After the relevance of the input coarse centroid set is computed, we calculate the weight of a centroid c as the relevance multiplied by the size of the centroid in terms of descriptors it stores. The weight is then used as the target metric to balance data partitioning instead of only the number of points employed in SABBS.

The SABBSR strategy is presented in Algorithm 6. It starts by computing the sum of weights for all coarse centroids, which is now the number of descriptors multiplied by the relevance (c.relevance()×c.size(),line3). Next, the threshold value is computed by dividing the sum of weights by the number of partitions (line 5). The centroids are clustered into n regions using the k-means algorithms based on their spatial location (line 6). Further, from line 10 to 23 the centroids are assigned to the target balanced regions. The iteration through the centroids takes place in a list of centroids sorted by their size (number of descriptors) in descending order. The reasoning for using this ordering is to allocate heavy-weighted partitions first to limit the imbalance from the allocation of the last centroids (the smallest ones) that may not fit any particular region when considering the threshold.

During the computation of the appropriate region to store a centroid, we first compute the n closest regions to a centroid c in line 11. Then, we search for the closest region where centroid c would fit without exceeding the threshold (see line 13). If a region can fit the centroid it is inserted in that partition (line 19), or it is inserted into its closest partition, even if exceeding the threshold (line 21). When all centroids are assigned to a region, the *balancedRegions* mapping is returned. Notice that the *sentTo* and *forward* operations used here are the same employed in SABES and SABBS, since only the grouping of data buckets changes among those strategies. Consequently, the computation complexity of the *sendTo* and *forward* is the same for SABES, SABBS, and SABBSR.

**Algorithm 6:** Bucket regions building with SABBSR.

1:	**function** constructRegion($C_{c},\;n$)
2:	**for** $c\in C_{c}$ **do**
3:	sizeVsRelevance←sizeVsRelevance+c.size()*c.relevance()
4:	**end for**
5:	threshold←sizeVsRelevance/n
6:	$\mathrm{kMeansRegions}\;\leftarrow \;\text{k-means}(C_{c},\;n)$
7:	**for each** i∈0…n **do**
8:	balancedRegions[i]←[]
9:	**end for**
10:	**for each** $c\;\in \;\text{sortedBySize}(C_{c})$ **do**
11:	closestRegions←k-NN(n,c,kMeansRegions.centroids)
12:	**for each** i∈0…n **do**
13:	**if** balancedRegions[closestRegions[i]].weight()+c.weight()≤threshold **then**
14:	r←i
15:	**break**
16:	**end if**
17:	**end for**
18:	**if** r<n **then**
19:	balancedRegions[closestRegion[r]].add(c)
20:	**else**
21:	balancedRegions[closestRegion[0]].add(c)
22:	**end if**
23:	**end for**
24:	**return** balancedRegions
25:	**end function**

## 6 Experimental Evaluation

This section presents the experimental evaluation performed to validate our proposed solutions. The experiments were conducted on a distributed memory environment with up to 60 compute nodes. Each node is equipped with a dual-socket Intel(R) Xeon(R) Gold 6252 CPU (48 cores per node), 360 GB of RAM, running Red Hat Enterprise Linux (RHEL) 7.9 with Open MPI 4.0.1. The dataset used in our experiments consists of up to 12 billion 128-dimensional SIFT descriptors and 100,000 queries created using images collected from the web [29]. For each query, the 5,000 closest neighbors were searched, defined as the parameter k. The search quality was evaluated using the commonly used recall@R metric that measures the proportion of query nearest neighbor ranked in the first R positions.

The IVFADC index has been configured with 4,096, 16,384, and 32,768 coarse centroids depending on the setup, where other parameters such as *w* were also varied. We fixed the number of subspaces used in the quantization process as *m* = 8 as it has also been shown to present a good performance vs. quality trade-off for SIFT descriptors in several previous works [5, 14, 30]. A comprehensive set of parameter configurations used in our experiments is presented in Table 2. Distributed memory execution efficiency has been evaluated on a weak scaling setup, with both computing resources and dataset size increasing proportionally. This metric is more relevant than strong scaling for the target domain because we are expected to index large and continuously growing datasets.

The remaining of this section is organized as follows: we first present the results of the Facebook AI Similarity Search (FAISS) library implementation [31] of the IVFADC algorithm, which is used as the baseline ANNS algorithm for parallelization here. Next, we experimentally present the limitations of the SABES strategy, which are addressed by this work. Further, we present a thorough evaluation of the state-of-the-art data partitioning strategies with a comparison to our newly proposed approaches.

## 6.1 IVFADC Search Performance

This section presents the compromises among the performance and quality of the IVFADC indexing algorithm. For that sake, we employed the FAISS library [31], an open-source solution for efficient similarity search. The intention of this section is only to demonstrate how efficient the approximate search can be and the typical trade-off associated with this class of algorithms. Furthermore, since IVFADC is used as our baseline algorithm for parallelization, we use its FAISS implementation in our parallel system when building the index locally in nodes of the distributed system and during the search. In this specific analysis, we have used 200 million SIFT descriptors with 4,096 centroids and a local machine equipped with an Intel(R) Core i5–1135G7 CPU with 16 GB of RAM.

**Figure 5:**
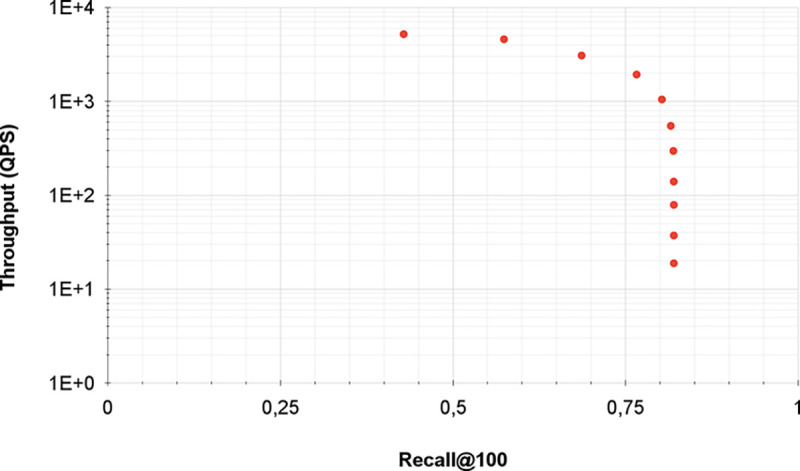
IVFADC throughput (queries processed per second - QPS) versus quality (Recall@100) using 200 million SIFT descriptors and 4,096 centroids in a sequential run. The throughout is presented in logarithmic scale.

Figure 5 demonstrates the relationship between throughput (queries processed per second) and Recall@100 using IVFADC. Each point represents a different configuration of IVFADC with an increasing value of w (the number of inverted file entries or coarse centroids visited to answer a query). As shown, IVFADC achieves high throughput while maintaining relatively good Recall@100 as w increases, which allows for the end-user of the indexing to configure it according to the desired compromise. For a detailed analysis of IVFADC, we refer the user to the previous works that have demonstrated its efficiency in several settings and compared it to other ANNS strategies [7, 31].

### 6.2 SABES Profiling: Understanding Data and Load Imbalance

This section presents the data imbalance observed in the SABES data partitioning and its correlation with load imbalance. These are the primary aspects that motivated the development of the data partitioning policies presented in this work. These aspects are investigated in a setting where a dataset with 300 million SIFT descriptors is distributed among 8 QP instances. IVFADC was configured to use 4,096 coarse centroids and 50,000 queries were executed on the system.

The number of data points stored in each QP instance using SABES is presented in Table 3 along with other metrics we have collected and discussed in this section. As may be seen, there is a significant discrepancy in the number of descriptors stored in each QP instance, as it ranges from 56 million to 22 million data points with a standard deviation of over 13 million. This aspect leads to imbalanced memory demands across QPs. This can be a significant limitation in homogeneous distributed machines because the minimum memory required is determined by the most demanding QP instance.

Furthermore, it is also critical to understand the impact of this data imbalance on the execution times of the QPs. As expected, this data imbalance also leads to load imbalance, resulting in significantly different execution times among QPs (see Table 3). Unfortunately, the distributed execution bottleneck is given by the slowest QP in the distributed system and, as a consequence, this negatively impacts the system performance. Finally, we highlight the variation in the average number of centroid accesses among QPs, which correlates directly with the relevance of the centroids within each QP, and such discrepancy suggests that load imbalance, and consequently runtime, will also be impacted by this aspect.

### 6.3 Performance Comparisons of Data Partitioning Strategies

This section evaluates the data partitioning strategies under different deployment and parameter configurations, which were enumerated in Table 2. SABBSR additionally requires the centroid relevance data for its indexing. This data was generated with 10,000 query vectors from a separate query dataset to perform the relevance profiling. Those queries were not used again during the runs. Section 6.3.1 investigates the effect of varying w (the number of coarse centroids accessed to answer a query) on the performance. In Section 6.3.2 we analyze the impact of varying the number of centroids, ranging from 4,096 to 32,768. Finally, Section 6.3.3 conducts the performance evaluation of partitioning strategies in a weak scaling. This comparison spans from a setup with 1 Coordinator and 4 Query Processors to a configuration with 12 Coordinators and 48 Query Processors. For all experiments, run time values are reported as the median over three runs, and a standard deviation smaller than 1% was observed across all experiments. Since any error bar would be too small to notice these were omitted.

#### 6.3.1 Performance Impacts of Varying w

This section evaluates the impact of w on the system performance for all data partitioning strategies evaluated: DES, BES, KD-Tree, SABES, SABBS, and SABBSR. In this experiment, we have used a fixed number of 40 nodes (8 Co, 32 QPs) with 8 billion SIFT descriptors, 16,384 centroids and, 100,000 queries. The following w values were analyzed: 8, 16, and 32.

**Figure 6:**
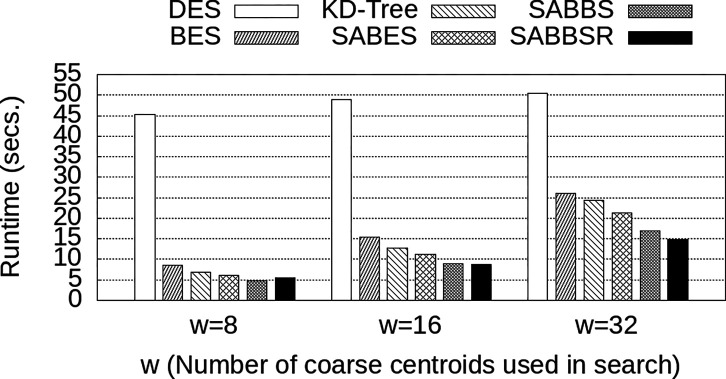
Execution time of different data partitioning strategies as the value of w is varied using 40 compute nodes.

The experimental results are presented in Figure 6. First of all, the execution time comparison of the data partitioning strategies demonstrates that our newly proposed approaches (SABBS and SABBSR) attained better performance regardless of the w value employed. In general, DES had the worst performance, followed by BES and KD-Tree. As observed in previous works, SABES has improved on top of DES, BES, and KD-Tree. Finally, SABBS and SABBSR attained superior performance compared to SABES, which is our main baseline. The actual speedups attained by SABBS and SABBSR on top of SABES are shown in Table 4, where it is more clear that significant gains were attained with a performance improvement of up to 1.43× with SABBSR.

**Figure 7:**
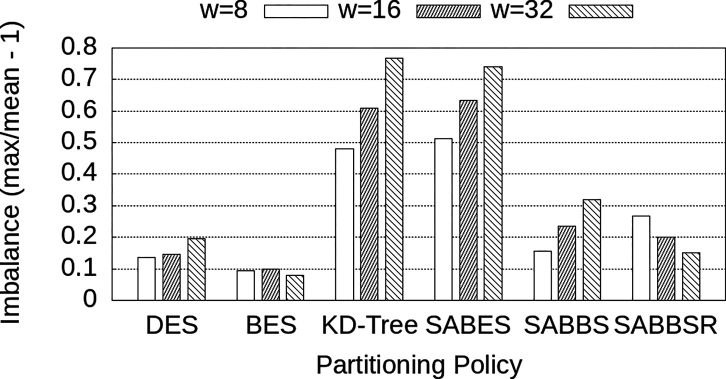
Runtime imbalance among QP instances for different data partitioning strategies and w values.

In order to better understand the differences in performance among data partitioning strategies, we have also measured the imbalance among QP instances observed in each case. The imbalance is computed here by dividing the execution time of the slowest QP by the average execution time minus 1. These results are presented in Figure 7. First, as expected, DES exhibits small imbalance values, since it partitions data equally among nodes. However, since this strategy requires every single QP instance to be involved in every query answer. This leads to larger data communication, overheads in the processing, and inefficiency in the search. Since BES distributes centroids or data buckets in a round-robin fashion, it also ends up balancing the load but does not benefit from data locality.

Further, SABES and KD-Tree exhibit the highest imbalance values across variations of the w parameter. This occurs because their regions are determined exclusively by spatial location, resulting in some regions (assigned to QPs) containing large datasets and execution times. When looking at SABBS and SABBSR, while those strategies benefit from spatial organization of the data buckets, their mechanisms to avoid data and load imbalanced allowed them to have a small to moderate imbalance, which is even comparable to DES and SABES which are expected to not suffer from this aspect. Finally, it was also interesting to observe that most strategies increase or maintain their imbalance rates as w grows, except for SABBSR. This happens because of the centroid relevance introduced by SABBSR. When a higher value of w is employed, the search examines a larger number of centroids per query, as a result, the relevance of the centroids becomes more important and SABBSR can better manage to reduce load imbalance.

#### 6.3.2 Performance Effects of Number of Coarse Centroids $\left(C_{c}\right)$

The number of coarse centroids used in IVFADC is another important parameter to be configured on IVFADC. Thus, this section evaluates the impact of modifying the number of coarse centroids used for the data partition strategies’ relative performance. This experiment also used a fixed number of 40 compute nodes (8 Cos and 32 QPs) with 8 billion SIFT descriptors and w=32. The configurations chosen for $C_{c}$ were: 4,096, 16,384, and 32,768 centroids. These are multiples of 4,096 typically used in the literature [7, 14, 31].

**Figure 8:**
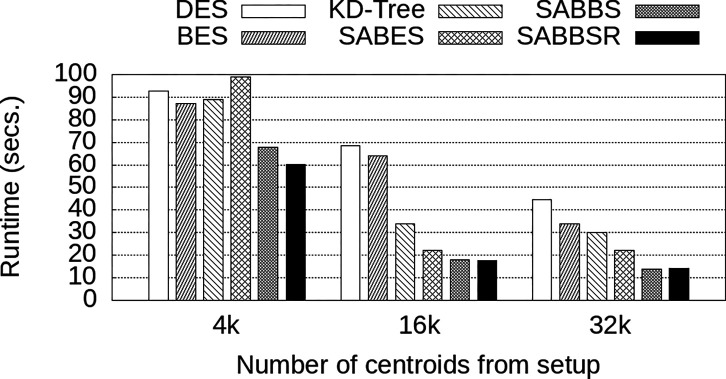
Execution time of different data partitioning strategies as the number of used coarse centroids $\left(C_{c}\right)$ is varied with a fixed number of 40 compute nodes.

The results are presented in Figure 8 and show that a larger number of centroids leads to improved runtime. This is expected because each centroid (bucket) will have fewer descriptors when the number of centroids grows. Thus, given that the number of searched centroids (w) per query is constant, the search is less expensive. Additionally, the ordering of strategies concerning performance is near to that observed in the last section, with the new SABBS and SABBSR improving on top of the previous work. Two aspects, however, deserve attention: (i) SABES achieved the worst performance for 4k centroids and (ii) the gains of SABBSR on top of SABBS decreased when a large number of centroids is used for a fixed w value. This first aspect is attributed to a high imbalance value of 1.4× with SABES for this particular configuration. The imbalance decreased to 0.69× and 0.33× for 16k and 32k coarse centroids. Given that the buckets distribution is imbalanced for SABES, larger buckets increase the potential for imbalanced data distribution, which explains its performance for $C_{c}=32,768$. The second observation is attributed to the diminishing relevance factor as the number of centroids increases without a corresponding growth in w. As such, it is expected that the SABBSR’s performance will become closer to SABBS’s. Finally, we summarize the gains attained by SABBS and SABBSR when compared to SABES in Table 5. We found gains of up to 1.64× with significant improvements for all configurations.

#### 6.3.3 Weak Scaling Evaluation

This section evaluates the data partition strategies on a weak scaling scenario in which the dataset and the available computing resources are increased in the rate. The number of nodes varies from 5 to 60, maintaining a ratio of 1 Co to each 4 QPs. Also, 100,000 queries were executed for IVFADC configured with 16,384 centroids and *w* = 64 while the dataset was scaled from 1 billion to 12 billion SIFT descriptors.

**Figure 9:**
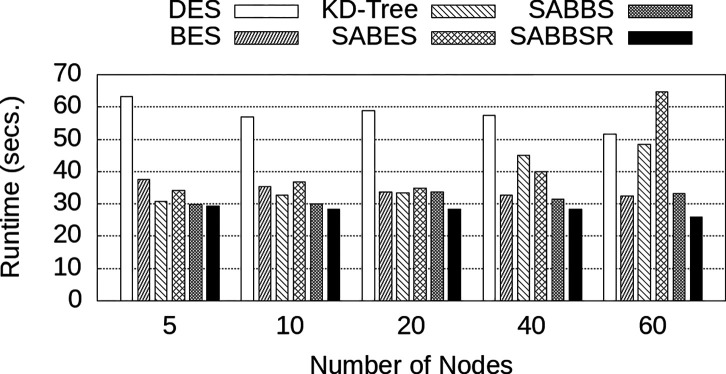
Runtime on a weak scaling evaluation using up to 60 compute nodes and 12 billion SIFT descriptors.

The runtime values are presented in Figure 9. It is noticeable that DES exhibited modest improvements as the number of nodes increased, which can be attributed to adding more coordinators. By increasing the number of coordinators while maintaining all other parameters fixed, the number of queries processed per second for each coordinator is smaller. This distribution reduces the time required for query reception and processing, contributing to an incremental improvement in performance.

**Figure 10:**
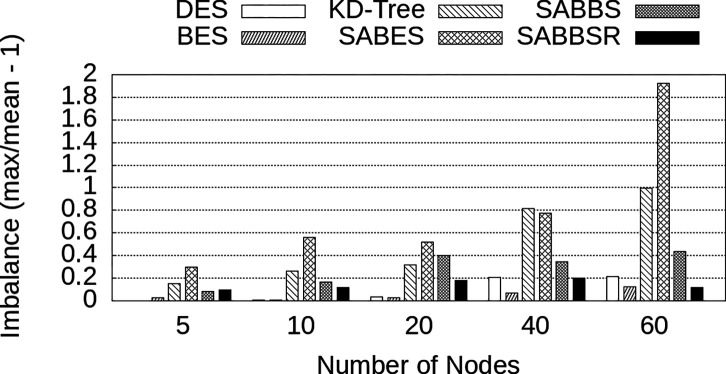
Imbalance of the weak scaling varying from 5 to 60 compute nodes with up to 12 billion SIFT descriptors.

Further, it can be seen that SABES only outperforms BES for a small number of nodes. However, as the number of nodes increases, SABES’s performance begins to suffer from the imbalance. Similarly, the KD-Tree strategy experiences imbalance, leading to a gradual increase in execution time. This aspect affects both SABES and KD-Tree as they give exclusive importance to the spatial locality in the partitioning, ignoring data balance among nodes. The imbalance for all strategies and node count configurations are shown in Figure 10. As shown, the decrease in performance of SABES and KD-Tree can be explained by a significant growth in imbalance as more nodes are employed. SABBS and SABBSR were able to better manage imbalance, with SABBSR achieving a much smaller imbalance index when compared to other approaches that organize data only using locality. These low imbalance values, combined with already present spatial locality led to improved performance at a larger scale.

Table 6 shows the average number of QPS used per query, representing the average count of QPS to which we forward the query to obtain the k nearest neighbors. This is one of the most important aspects of spatial data organization. It demonstrates the efficiency of spatial-aware strategies in maintaining bucket locality in QPs. DES needs to access all QPs at each query, thus, this value is the same as the number of QPs used. For other strategies, a smaller number of QPs may be accessed depending on the data organization. Since BES distributes centroids in a round-robin fashion, its results are closer to DES. However, the other policies that explicitly consider spatial locality tend to benefit in that respect. For instance, SABES which has the best locality organization uses on average between 2 and 8 QPs to answer a query. In contrast, DES requires accessing over 5× more QPs than SABES. These results also portray that the best bucket locality comes at the cost of imbalance. This trade-off is leveraged by SABBS and SABBSR to improve runtime performance while maintaining a low number of QPs accessed by query. Moreover, the number of QPs used grows sub-linearly for both SABBS and SABBSR.

Finally, Table 7 summarizes the comparison of the data partitioning strategies in terms of speedup using BES as a baseline. BES was chosen here because it is the best non-spatial aware strategy in the literature. As may be seen, SABBSR is the best-performing approach and it has achieved consistent performance improvements on top of BES regardless of the configuration used with an average speedup of 1.22×.

## 7 Conclusions and Future Directions

This work focused on the problem of partitioning databases for efficient execution of similarity search on distributed memory computing systems. We have revisited state-of-the-art partitioning strategies, presenting and discussing their limitations in scale. In particular, spatial-aware data partitioning approaches have been shown to attain significant performance gains compared to more traditional strategies, such as DES and BES, in particular scenarios. However, they have shown limited improvements in other cases mainly due to load/data imbalance that can arise with their partitioning.

To address the limitations of previous strategies we have proposed two new data partitioning strategies: SABBS and SABBSR. These strategies are compared with the baseline DES, BES, KD-Tree, and SABES in various configurations, where it was shown that our approaches have significantly improved the state-of-the-art approaches. For instance, SABBS and SABBSR achieved speedups of up to 1.60× and 1.64×, respectively, on top of SABES when the number of coarse centroids used was varied. Also, as w is varied, SABBS and SABBSR improved on SABES in up to 1.25× and 1.43×, respectively.

Further, a large-scale weak scaling evaluation with up to 60 nodes has demonstrated that the performance gains were also observed in scale while indexing a dataset with up to 12 billion descriptors. The performance gains of the SABBS and SABBSR strategies result from improvements in data partitioning imbalance, which translated to a reduced runtime imbalance among Query Processors (QPs) and, consequently, a smaller application makespan.

This paper has also opened the avenue for several future works. We first want to expand the analysis of the data partitioning strategies considering other indexing algorithms. While IVFADC has been known to work well with large datasets while maintaining a relatively low memory footprint, there are specific scenarios in which other ANNS indexing solutions are also competitive. Thus, understanding if the compromises observed here will hold for other indexing algorithms is an important milestone to consolidate this area in the similarity search domain. Further, we would like to analyze how the strategies proposed here could be evolved to deal with heterogeneous computing platforms, such as CPU and GPU machines or clustered distributed machines with different CPU and memory configurations. Both types of heterogeneity are becoming more frequent, for instance, in cloud-based machines.

## Figures and Tables

**Table 1:** Comparative table for distributed ANNS related works.

Name	ANNS Approach	Index Location	Distributed Data Partitioning Characteristics		

			Data-Balancing Aware	Spatial Aware	Load-Balancing Aware

PLSH [20]	LSH	In-memory	✗	✗	✗
SES-SLH [21]	LSH	In-memory	✗	✓	✗
RDH [22]	LSH	In-memory	✓	✗	✗
FLANN [23]	Graph	In-memory	✓	✗	✗
Pyramid [24]	Graph	In-memory	✓	✗	✗
SPANN [25]	PQ	Hybrid	✓	✗	✗
SABES [14]	PQ	In-memory	✗	✓	✗
This Work	PQ	In-memory	✓	✓	✓

**Table 2:** Dataset configuration and IVFADC parameters evaluated through the experiments.

Parameter	Value

N^o^ Coordinators	up to 12
N^o^ Query Processors	up to 48
Coarse Centroids	up to 32,768
N^o^ Inverted Lists accessed (*w*)	up to 64
Descriptors Dimension	128
Descriptors Type	SIFT
Dataset size	up to 12 Billion
Number of nearest neighbors (k)	5,000
Number of Queries	100,000

**Table 3:** Profile of the SABES using 8 QPs. The metrics demonstrate the observed data imbalance, its impact on the execution times, and the number of centroid accesses, which directly impacts the relevance attributed to it.

QP ID	Time	Number of Descriptors	Number of Centroids	Average # of Centroid Accesses

7	254.02	45,983,692	510	243.58
4	202.62	30,353,644	414	210.93
5	294.05	56,369,612	863	176.47
6	204.61	29,854,512	379	187.88
2	197.18	28,185,376	409	188.01
1	211.29	31,287,980	457	194.65
3	175.08	22,108,128	278	211.03
0	326.88	55,857,052	786	178.67

**Table 4:** Speedups of SABBS and SABBSR when compared to SABES for different values of *w*.

Speedup against SABES	w=8	w=16	w=32

SABBS	1.25×	1.26×	1.25×
SABBSR	1.10×	1.28×	1.43×

**Table 5:** SABBS and SABBSR speedups on top of SABES for a different number of coarse centroids (*C_c_*) and *w* = 32.

	4k	16k	32k

SABBS	1.46×	1.24×	1.60×
SABBSR	1.64×	1.25×	1.55×

**Table 6:** Average number of QPs accessed to answer a query on a weak scaling.

Partitioning Policy	Number of Nodes				

	5	10	20	40	60

**DES**	4	8	16	32	48
**BES**	4	7.99	15.75	27.88	35.57
**KD-Tree**	3.04	5.24	8.59	13.45	17.15
**SABES**	2.08	3.21	4.83	6.84	8.22
**SABBS**	3.15	5.07	6.79	9.64	12.03
**SABBSR**	3.04	4.87	6.68	9.73	12.27

**Table 7:** Speedup over BES for all strategies in weak scaling.

Partitioning Policy	Number of Nodes				

	5	10	20	40	60

**KD-Tree**	1.22×	1.07×	1.00×	0.72×	0.66×
**SABES**	1.10×	0.95×	0.96×	0.81×	0.50×
**SABBS**	1.26×	1.17×	0.99×	1.04×	0.97×
**SABBSR**	1.28×	1.23×	1.18×	1.15×	1.25×
